# Opportunities and Challenges Associated with the Uptake of Residential Clean Fuel Usage

**DOI:** 10.1007/s40572-024-00438-7

**Published:** 2024-03-12

**Authors:** Darpan Das, Sohail Ahmad, Joshua Kirshner

**Affiliations:** Department of Environment and Geography, University of York, Heslington, York YO10 5NG UK

**Keywords:** Cooking, Air Pollution, Exposure

## Abstract

**Purpose of Review:**

Almost 3 billion people worldwide use solid fuel for cooking and heating. This review examines (i) household energy practices and infrastructures and their influence on fuel usage in different contexts; (ii) barriers in adoption of household clean energy technologies and uses in diverse settings and population groups and (iii) potential air pollution exposure reduction in homes through using processed fuel.

**Recent Findings:**

Population health burden from solid fuel combustion-derived particulate air pollution has been estimated in several low- and middle-income countries. However, such studies have not been carried out in high income countries (e.g., UK). Irrespective of the region, fuel prices are the most dominant factor influencing the choice of fuel. Laboratory studies suggest processed fuel — pellets and briquettes — reduce particulate matter emissions by 70–80% and can be a promising alternative.

**Summary:**

Adoption of clean fuels for domestic energy needs facilitates progress towards five of the UN Sustainable Development Goals (SDGs). There is evidence that a variety of factors, including cost savings, encourage and hinder such uptake. These factors include price fluctuations, expenses, and the usage of clean fuels. Due to their distinct development scenarios, more expansive policy frameworks, and political economies of energy, these determinants are localized in character and differ significantly amongst economies. Therefore, in order to create innovative plans for the adoption of clean fuel use, strategies centred on local settings must be developed while keeping broad socio-technical and socio-economic issues in mind. Solid fuel processing — pelletization and briquetting — have the potential to reach Liquefied Petroleum Gas (LPG)–like emissions, and could be a potential strategy to mitigate exposure to household air pollution

## Introduction

Household air pollution is one of the more prominent forms of air pollution that are known to be associated with long-term health effects of air pollution — lung cancer, respiratory disease and chronic obstructive pulmonary diseases [1, 2]. Approximately 0.31 million lung cancer deaths and 7.02 million disability adjusted life years occurred globally in 2019 due to exposure to ambient PM_2.5_ [3]. In global south countries alone, the economic losses due to premature deaths as a result of use of solid-fuel use amounts to US$2.4 trillion annually [4]. Household air pollution is typically associated with the burning of biomass and coal for cooking and heating [5]. Incomplete combustion of wood and coal leads to the formation of several pollutants — carbon monoxide (CO), carbon dioxide (CO_2_), methane (CH_4_), particulate matter (PM), nitrogen oxides, volatile organic compounds, dioxins, furans, precursors of ozone, and secondary organic aerosols, which impact air quality and human health [6].

While solid fuel usage for cooking needs is visible in low-and-middle income countries located in the global south; its usage for heating needs has been documented in several countries of the global north. Combustion emissions cause ∼13,000 premature deaths in the UK per year [7]. Tomlin, 2021 [6] identified that biomass smoke contributes to at least 40,000 premature deaths per year in Europe, as well as negatively affecting respiratory and cardiovascular health. Notably, socio-spatially disadvantaged communities are disproportionately impacted across both the global North and South [8, 9]. For instance, Ferguson et al. [8] using a case study of London, found evidence of five factors resulting in disproportionately higher indoor air pollution exposure amongst disadvantaged communities: housing location and ambient outdoor levels of pollution; housing characteristics including ventilation properties and internal sources of pollution; occupant behaviours; time spent indoors; and underlying health conditions. Given its universal context, the UN’s SDG emphasizes the need to “ensure access to affordable, reliable and modern energy for all by 2030” [10]. International agreements on sustainable energy access (SDG 7) and climate change are mobilizing billions of dollars in finance for technology transfer to the Global South. This has led to an increase in generation capacity and extension in grid infrastructures for household connections. Apart from SDG 7, cooking/heating with clean fuels supports SDG Goal 3 (Good health and wellbeing), Goal 5 (gender equality), and Goal 15 (Life on land) [4]. In the present study, we focussed on research published within the past 5 years on the three themes of (a) large scale energy transition, (b) barriers and adoption, and (c) processed fuel.

## The Large-Scale Energy Transition

In the recent past, the use of LPG as a primary cooking fuel in India has been tremendously successful — it has risen from merely 33% in 2011 to 71% in 2020, according to the Indian Residential Energy Consumption Survey conducted by the Council on Energy, Environment and Water (CEEW) [11]. The national government has provided nearly 100 million new households with LPG connections under the *Pradhan Mantri Ujjwala Yojana* (PMUY) since 2016 (ibid). However, recent global events such as COVID-19 have dented universal use of LPG in Indian households; for instance, estimates have revealed a 0.5% decrease in the use of LPG in FY 23 from FY 22. In order to achieve universal use of LPG, still major barriers are switching and running costs, resistance to adoption of new technology, and cultural associations with food cooked with three stone stoves [12]. Currently, LPG is seen as the only means to achieve India’s clean-cooking energy, a programme that has sought to diversify fuel sources, such as the use of electricity as a cooking fuel. Currently, about 10% of households in urban India use electricity as their primary cooking fuel that can offset flame-based cooking as a result of calibrating existing fuel subsidies.

While large scale clean^1^ cooking programmes in Asia — particularly India and China, have been successful in reducing household air pollution, such implementation schemes have not been particularly prominent in Africa [14]. Almost 1 billion people in sub-Saharan Africa still use dirty fuel for their cooking needs and it is expected that this limited access to clean cooking fuels will continue till 2050. While pellets and briquettes are not typically used in Mozambique, charcoal is used widely across urban and rural areas. In Maputo, Mozambique’s capital city of nearly 2 million, research led by Castán Broto [15] found that residents use diverse fuels for cooking and heating. The pattern of charcoal as the primary fuel of choice in the city is losing ground, however, due to precarities in charcoal production and distribution, a growing middle-class with changing consumption patterns, and the evolving nature of fuel poverty [15]. Researchers have estimated that roughly 80% of urban residents in Mozambique consume charcoal [16]. Yet, those using charcoal exclusively drops to 50% in Maputo city [17]. In Maputo, this shift has been supported through the increased availability of LPG and electricity grid connections provided by the national power utility. LPG imports have increased in Mozambique from 5000 tonnes in 1990 to 24,000 tonnes in 2014 (IEA, 2014). Further, EDM figures suggest that over 91.9% of households in Maputo were connected to the national electricity grid by 2015 [18].

Despite this growing availability of alternate sources, charcoal persists in Mozambique’s cities, shaping urban spaces and social relations. It is visibly traded, often in small, affordable quantities, in market stalls and on street corners across Maputo and other larger and smaller cities, in places where LPG sales and supporting infrastructures frequently do not reach [19]. As in other countries highly dependent on biomass fuels, households in Maputo frequently engage in fuel ‘stacking,’ or partially adopting clean fuels such as electricity and LPG, but without fully replacing traditional or subsistence fuels, such as charcoal and fuelwood [20, 21]. This suggests a strategy of multiple and overlapping fuel usage that remains prevalent across different sites and social groupings [15].

As the Mozambican example suggests, the expansion of residential electricity grid connections does not automatically guarantee access to reliable and sustainable electricity. The choice of energy source within households has influenced a range of factors, including grid reliability and affordability, social behaviours and everyday practices of energy use in domestic settings, along with fluctuating and uncertain prices for both electricity and charcoal, and on households’ capability to invest in new energy-consuming appliances [22, 23]. Conversely, biomass sources and kerosene are often more expensive per unit of useful energy than higher-grade sources, suggesting that although electrification has proved challenging, it continues to be an effective means for poverty alleviation [15, 24].

## Barriers and Adoption

Eakins et al. [25] in their study in Ireland have identified geographical location, household income and fuel cost as influencing the user towards sourcing fuel from the informal sector as compared to the formal sector.^2^ Irrespective of geographical location, pricing was considered as the most significant barrier for switching to an efficient technology [26, 27]. Ahmad & De Oliveira [26] found factors such as fuel prices, access to electricity and water supply, and education attainment influence the type of fuel used in India.

In the UK, building design can be related with fuel poverty, as these buildings will have structures with open fires [28]. Tweed et al. [29] identified that people usually prefer radiant heat and a living flame. Noise pollution from heat pumps is also an issue for several users. These measures may encompass primary actions like regulating fuel quality, implementing educational programmes on proper device usage, and incorporating designs that minimize improper operation. Additionally, secondary measures, such as catalytic removal methods, could also be considered.

Better understanding of the pollution generated by biomass burning and its associated health consequences could contribute to encouraging behavioural shifts [6]. Tomlin [6] also pointed out the challenge of envisioning legislation that significantly restricts households’ options to burn solid fuels.

Availability and affordable cost are the usual barriers to large scale adoption of clean energy — LPG [30]. While most household air pollution studies have primarily focussed on the developing world, there are limited studies which quantify the usage of solid fuel in high-income countries. Air pollution is the greatest environmental public health threat in the UK with 29,000—43,000 deaths annually [31]. Further, estimates suggest that 13% of families in England (~ 3.5 million households), 25% in Scotland, 14% in Wales and 24% in Northern Ireland live in fuel poverty,^3^ with numerous households potentially shifting to charcoal-based or wood burning stoves to save energy costs. Such a move could lead to an unprecedented volume of solid fuel being used for domestic heating, leading to hyperlocal hotspots of air pollution. Further, with the advent of higher energy costs, residents are expected to start burning more solid fuel in their homes, as opposed to using gas-based central heating [32].

Most houses in the UK are equipped with modern and energy efficient forms of domestic cooking. However, fossil fuels remain the main source of space heating in the UK. More than 50% of renewable heat generation in the residential sector in the UK is from wood combustion [33]. Residential solid fuel usage in UK homes typically comprises of peat, wood and coal [34, 35]. Alternatively, heat pumps, a key renewable energy source, could be used for heating purposes. Sadly, the UK has relatively low deployment of heat pumps, as compared with neighbouring European countries such as France and Germany [36, 37]. In contrast to the other European countries, the UK’s historical reliance on cheap gas and poor quality of housing stocks have not enabled the uptake of heat pumps. Based on a synthesis review, Ahmad [36] identified the motivations and barriers to uptake of heat pumps in the UK broadly covering three themes: households’ socio-technical characteristics, built environment attributes and stakeholders’ competing and differing economic and organisational interests (Table 1). Principal evidence suggests that saving money, increasing household energy independence, and reducing greenhouse gas emissions are the positive drivers to adopt heat pumps whereas higher capital and running costs are the major barriers. The current imbalance in energy taxes and levies (environmental levies are multiple folds higher on electricity than gas) further weakens the economic competitiveness of heat pumps in the UK. Furthermore, appropriate knowledge and awareness, including about choices, technical and financial, helps the adoption of heat pumps in the UK.

**Table 1:** Motivations for and barriers to adopting for clean fuels for domestic usage

	Motivation	Barrier
Financial	- Reducing fuel bills (in selected options) - Protecting against future high energy costs	- Capital costs and running costs of clean fuels (e.g., solid cooking fuel to LPG) - Freely available alternative energy sources (e.g., solid cooking fuels in suburbs) - Poor socio-economic bases
Environmental	- Desire to reduce carbon emissions and indoor pollution	- Often environmental benefits are perceived as too small
Knowledge and awareness	- Community-based plans and champions	- Perceived complexity of the technology - Lack of trustworthy information - Lower educational attainment
Miscellaneous	- Increasing household energy independence	- Existing building design (sometimes not suitable for new modern technologies) - Geographic location (e.g., off-grid locations face difficulty to gas-based boilers)

Source: Adopted from various sources, including Ahmad [36]

## Mitigation Using Processed Fuel

More than 50% of renewable heat generation in the residential sector in the UK is from wood combustion [33]. Current clean energy sources (e.g., heat pumps) in the UK are insufficient to meet residential space heating demands [38]. Rising energy bills and economic recession have led residents of the UK to resort to the usage of heating stoves. Although, the UK Government is setting an annual PM_2.5_ mean concentration limit of 10 µg/m^3^, high usage of heating stoves may lead to non-compliance. The use of bioenergy with carbon capture and sequestration technology to offset some greenhouse gas emissions can be a potential solution in decarbonising the UK’s residential energy sector.

Processed fuel (torrefied and briquetted) has been identified as a promising carbon neutral source, and has reduced emissions relative to raw fuel [39, 40]. Das et al. in their study found that carbonization^4^ of coal can lead to approximately 70% reduction in emissions [41]. However, these volatiles are an important contributor to the initial ignition stage [42] and removal of them leads to a higher ignition temperature [41, 43]. Briquettes with locally available binders help in initial ignition and thus lead to reduction of overall mass-based particulate matter emissions [44]. Mitchell et al. [33] found that renewable briquettes used in cooking stoves led to lower emissions as compared with their raw form and can be a promising alternative. Das et al. [44] demonstrated that briquettes have an ability in decreasing PM_2.5_ emissions with potential of 45–65%. Net climate impacts of pellet stoves are also similar to LPG [45]. Pradhan et al. [46] in their study on developing a conceptual framework for a bio-energy system for rural India identified a pellet-based gasifier system to be cost competitive with commercial liquefied petroleum gas (LPG).

Fuel treatment methods involve removing moisture [47], semi volatiles [48], and inorganic components [49] from the fuel. Large scale treatment of fuel could be a potential solution; however, these emissions from treatment plants themselves need to be properly regulated using adequate air pollution control device. Moreover, the extensive adoption of biomass on a large scale would strain supply chains, necessitating the utilization of carbon–neutral sources involving sustainable replanting or genuinely waste materials [6]. Attaining global net zero greenhouse gas (GHG) emissions demands the implementation of bioenergy with carbon capture and sequestration technology.

## Conclusions

The impacts of air pollution are unequal with the most disadvantaged communities exposed to the most polluted air. Air pollution in the megacities of the global south is frequently several folds higher than in the cities in global north. The uptake of clean domestic energy, including cooking fuels, has tremendous potential to mitigate exposure to indoor air pollution, along with systemic inequalities that drive this exposure. Processing of fuel has been found to be an efficient clean energy source. Evidence suggests that numerous factors provide motivations for such uptake, such as cost savings, and barriers to uptake, such as fluctuating prices, the use of clean fuels. These factors are localised in nature and vary substantially from one economy to another given their differentiated development scenarios and broader policy frameworks and political economies of energy. Accordingly, there is a need for developing strategies based on local contexts keeping broad socio-technical and socio-economic aspects in mind to develop creative plans for the uptake of clean fuel use.

## Footnotes

1 Energy sources from which carbon emissions are negligible or zero are termed clean energy. In contrast, energy sources with significant carbon emissions are termed dirty energy [13].

2 Eakins et al., [25] defines formal sector as legal commercial suppliers and fuel merchants, where solid fuels can be purchased from. Informal sectors are grey markets from unregistered traders e.g., private sellers such as farmers or landowners, free off one’s own land through the collection of foraged wood or wind-blown trees, harvesting of peat bogs and supply of sod peat is another commonly used indigenous source of solid fuel energy to many households that own or rent peat bogs.

3 Fuel poverty is defined differently in different parts of the UK as it is the part of the devolved policy area. “In general, fuel poverty relates to households that must spend a high proportion of their household income to keep their home at a reasonable temperature”. (Parliament. House of Commons, 2023, p.4) https://researchbriefings.files.parliament.uk/documents/CBP-8730/CBP-8730.pdf

4 Carbonization is the process of heating in the absence of oxygen, thus removing the moisture and volatile content within the fuel [41].
